# Supplementary material for: Allosteric regulation and crystallographic fragment screening of SARS-CoV-2 NSP15 endoribonuclease
Source: Nucleic Acids Res. 2023 Apr 28;51(10):5255–70. doi: 10.1093/nar/gkad314 (PMC10250223; doi:10.1093/nar/gkad314)
Supplement: gkad314_Supplemental_Files [file gkad314_supplemental_files.zip › Supplementary datasets.zip!/183/Refine_4/01-BUSTER/Cycle-5/shell.01/R_Rfree.html]

# Rxpct factors in resolution bins at cycle 0


|  |  |  |  |  |  |  |  |  |
| --- | --- | --- | --- | --- | --- | --- | --- | --- |
|  | **<d> (Å)**  Rxpct\_Work | Rxpct\_Free | Rxpct\_All | |  |  |  |  |
|  | 14.0455 | 0.3208 | 0.3714 | 0.3228 |  |
|  | 8.1092 | 0.2247 | 0.2941 | 0.2288 |  |
|  | 6.2814 | 0.2347 | 0.2801 | 0.2369 |  |
|  | 5.3087 | 0.1942 | 0.2300 | 0.1959 |  |
|  | 4.6818 | 0.1708 | 0.1958 | 0.1721 |  |
|  | 4.2349 | 0.1730 | 0.1724 | 0.1730 |  |
|  | 3.8955 | 0.1776 | 0.2117 | 0.1792 |  |
|  | 3.6265 | 0.1898 | 0.2429 | 0.1925 |  |
|  | 3.4065 | 0.1859 | 0.2057 | 0.1870 |  |
|  | 3.2223 | 0.1843 | 0.2110 | 0.1858 |  |
|  | 3.0650 | 0.1831 | 0.1927 | 0.1836 |  |
|  | 2.9287 | 0.1831 | 0.2043 | 0.1841 |  |
|  | 2.8091 | 0.1864 | 0.2120 | 0.1875 |  |
|  | 2.7031 | 0.1931 | 0.2152 | 0.1943 |  |
|  | 2.6082 | 0.2055 | 0.2244 | 0.2066 |  |
|  | 2.5227 | 0.2069 | 0.2321 | 0.2084 |  |
|  | 2.4450 | 0.2103 | 0.2349 | 0.2115 |  |
|  | 2.3741 | 0.2247 | 0.2346 | 0.2251 |  |
|  | 2.3091 | 0.2518 | 0.2830 | 0.2531 |  |
|  | 2.2491 | 0.3042 | 0.3098 | 0.3044 |  |
|  | **Overall** | 0.2000 | 0.2275 | 0.2014 |  |

# Rxpct factors in resolution bins at cycle 1


|  |  |  |  |  |  |  |  |  |
| --- | --- | --- | --- | --- | --- | --- | --- | --- |
|  | **<d> (Å)**  Rxpct\_Work | Rxpct\_Free | Rxpct\_All | |  |  |  |  |
|  | 14.0455 | 0.3209 | 0.3716 | 0.3229 |  |
|  | 8.1092 | 0.2248 | 0.2943 | 0.2289 |  |
|  | 6.2814 | 0.2346 | 0.2805 | 0.2369 |  |
|  | 5.3087 | 0.1941 | 0.2301 | 0.1958 |  |
|  | 4.6818 | 0.1706 | 0.1959 | 0.1720 |  |
|  | 4.2349 | 0.1727 | 0.1721 | 0.1727 |  |
|  | 3.8955 | 0.1772 | 0.2116 | 0.1788 |  |
|  | 3.6265 | 0.1895 | 0.2427 | 0.1922 |  |
|  | 3.4065 | 0.1857 | 0.2056 | 0.1867 |  |
|  | 3.2223 | 0.1840 | 0.2108 | 0.1855 |  |
|  | 3.0650 | 0.1830 | 0.1927 | 0.1834 |  |
|  | 2.9287 | 0.1830 | 0.2046 | 0.1840 |  |
|  | 2.8091 | 0.1863 | 0.2119 | 0.1875 |  |
|  | 2.7031 | 0.1930 | 0.2153 | 0.1942 |  |
|  | 2.6082 | 0.2054 | 0.2244 | 0.2065 |  |
|  | 2.5227 | 0.2068 | 0.2320 | 0.2082 |  |
|  | 2.4450 | 0.2101 | 0.2344 | 0.2112 |  |
|  | 2.3741 | 0.2244 | 0.2347 | 0.2249 |  |
|  | 2.3091 | 0.2513 | 0.2828 | 0.2527 |  |
|  | 2.2491 | 0.3045 | 0.3098 | 0.3047 |  |
|  | **Overall** | 0.1998 | 0.2275 | 0.2012 |  |

# Rxpct factors in resolution bins at cycle 2


|  |  |  |  |  |  |  |  |  |
| --- | --- | --- | --- | --- | --- | --- | --- | --- |
|  | **<d> (Å)**  Rxpct\_Work | Rxpct\_Free | Rxpct\_All | |  |  |  |  |
|  | 14.0455 | 0.3209 | 0.3718 | 0.3229 |  |
|  | 8.1092 | 0.2248 | 0.2944 | 0.2289 |  |
|  | 6.2814 | 0.2344 | 0.2810 | 0.2367 |  |
|  | 5.3087 | 0.1937 | 0.2302 | 0.1954 |  |
|  | 4.6818 | 0.1703 | 0.1960 | 0.1717 |  |
|  | 4.2349 | 0.1723 | 0.1717 | 0.1723 |  |
|  | 3.8955 | 0.1767 | 0.2113 | 0.1784 |  |
|  | 3.6265 | 0.1891 | 0.2424 | 0.1918 |  |
|  | 3.4065 | 0.1854 | 0.2054 | 0.1864 |  |
|  | 3.2223 | 0.1837 | 0.2107 | 0.1853 |  |
|  | 3.0650 | 0.1828 | 0.1927 | 0.1832 |  |
|  | 2.9287 | 0.1828 | 0.2049 | 0.1838 |  |
|  | 2.8091 | 0.1862 | 0.2117 | 0.1874 |  |
|  | 2.7031 | 0.1928 | 0.2154 | 0.1941 |  |
|  | 2.6082 | 0.2054 | 0.2244 | 0.2065 |  |
|  | 2.5227 | 0.2066 | 0.2319 | 0.2081 |  |
|  | 2.4450 | 0.2100 | 0.2340 | 0.2111 |  |
|  | 2.3741 | 0.2243 | 0.2347 | 0.2248 |  |
|  | 2.3091 | 0.2511 | 0.2825 | 0.2525 |  |
|  | 2.2491 | 0.3048 | 0.3098 | 0.3050 |  |
|  | **Overall** | 0.1995 | 0.2274 | 0.2010 |  |

# Rxpct factors in resolution bins at cycle 3


|  |  |  |  |  |  |  |  |  |
| --- | --- | --- | --- | --- | --- | --- | --- | --- |
|  | **<d> (Å)**  Rxpct\_Work | Rxpct\_Free | Rxpct\_All | |  |  |  |  |
|  | 14.0455 | 0.3142 | 0.3727 | 0.3165 |  |
|  | 8.1092 | 0.2246 | 0.2956 | 0.2288 |  |
|  | 6.2814 | 0.2295 | 0.2924 | 0.2326 |  |
|  | 5.3087 | 0.1879 | 0.2277 | 0.1897 |  |
|  | 4.6818 | 0.1670 | 0.2039 | 0.1691 |  |
|  | 4.2349 | 0.1694 | 0.1783 | 0.1699 |  |
|  | 3.8955 | 0.1740 | 0.2170 | 0.1760 |  |
|  | 3.6265 | 0.1854 | 0.2394 | 0.1881 |  |
|  | 3.4065 | 0.1824 | 0.2040 | 0.1836 |  |
|  | 3.2223 | 0.1816 | 0.2141 | 0.1834 |  |
|  | 3.0650 | 0.1807 | 0.1895 | 0.1811 |  |
|  | 2.9287 | 0.1822 | 0.2051 | 0.1832 |  |
|  | 2.8091 | 0.1855 | 0.2176 | 0.1869 |  |
|  | 2.7031 | 0.1932 | 0.2180 | 0.1946 |  |
|  | 2.6082 | 0.2054 | 0.2231 | 0.2065 |  |
|  | 2.5227 | 0.2063 | 0.2409 | 0.2082 |  |
|  | 2.4450 | 0.2092 | 0.2329 | 0.2103 |  |
|  | 2.3741 | 0.2243 | 0.2339 | 0.2248 |  |
|  | 2.3091 | 0.2519 | 0.2962 | 0.2539 |  |
|  | 2.2491 | 0.3049 | 0.3094 | 0.3051 |  |
|  | **Overall** | 0.1971 | 0.2302 | 0.1987 |  |
